# A 45-bp insertion/deletion polymorphism of UCP2 gene is associated with metabolic syndrome

**DOI:** 10.1186/2251-6581-13-12

**Published:** 2014-01-07

**Authors:** Mohammad Hashemi, Hamzeh Rezaei, Mahmoud-Ali Kaykhaei, Mohsen Taheri

**Affiliations:** 1 Cellular and Molecular Research Center, Zahedan University of Medical Sciences, Zahedan, Iran; 2 Department of Clinical Biochemistry, School of Medicine, Zahedan University of Medical Sciences, Zahedan, Iran; 3 Department of Internal Medicine, School of Medicine, Zahedan University of Medical Sciences, Zahedan, Iran; 4 Genetics of Non Communicable Disease Research Center, Zahedan University of Medical Sciences, Zahedan, Iran

**Keywords:** Metabolic syndrome, UCP2, Polymorphism, Insertion/deletion

## Abstract

**Background:**

Metabolic syndrome (MeS) is being recognized as a risk factor for insulin resistance and cardiovascular disease. The present study was aimed to find out the possible association between 45-bp I/D polymorphism of uncoupling protein 2 (UCP2) and MeS.

**Methods:**

DNA was extracted from peripheral blood of 151 subjects with and 149 subjects without MeS. 45-bp I/D variant of UCP2 was detected using polymerase chain reaction (PCR).

**Results:**

Our finding showed that 45-bp I/D polymorphism was associated with protection against MeS (OR = 0.56, 95% CI = 0.34-0.92, p = 0.020 D/I vs DD and OR = 0.54, 95% CI = 0.34-0.86, p = 0.009; D/I + I/I vs D/D). The I allele decreased the risk of MeS (OR = 0.62, 95% CI = 0.44-0.90, p = 0.011) in comparison with D allele.

**Conclusion:**

In conclusion, our result suggests that 45-bp I/D polymorphism is associated with the risk of MeS, which remains to be cleared.

## Introduction

Metabolic syndrome (MeS) is described as the combination of clinical disorders that increase the risk for obesity (central adiposity), insulin resistance, glucose intolerance, dyslipidemia, non-alcoholic fatty liver disease and cardiovascular diseases including atherosclerosis, stroke and hypertension [1,2]. The prevalence of MeS is considerably increasing globally, and is becoming an important health problem [3]. The etiology of this syndrome is complex and is thought to be the result of interaction between genetic and environmental factors [4-6]. Uncoupling proteins (UCPs) are mitochondrial membrane transporters that disturb the coupling between the mitochondrial proton gradient and ATP synthesis. In humans, the gene for UCP2 is located on chromosome 11q13 and contains 8 exons which exons 1 and 2 of UCP2 are untranslated [7]. Among the five UCP homologs (UCP1–UCP5), UCP2 is the most widely expressed, being involved in thermal regulation in various tissues, including white adipose tissue, liver, kidney, pancreatic islets, macrophages as well as retinal endothelial cells [8,9], and it is thought to play a role in the progress of obesity [10]. This protein uncouples oxidation of substrate from phosphorylation, dissipating the membrane potential energy and consequently decreasing ATP production by the mitochondrial respiratory chain [8]. It has been proposed that uncoupling leads to tissue-specific functions such as decreasing ROS formation by mitochondria, regulation of free fatty acids metabolism and inhibition of insulin secretion from beta cells [8,11]. Increased ROS production has been observed in macrophages [12] and pancreatic islets of UCP2 knockout mice [13]. It has been reported that overexpression of UCP2 attenuates ROS production and prevents oxidative damage of tissues [14,15]. α-Cells of pancreas secrete glucagon in response to low blood glucose. It has been shown that UCP2 is required for appropriate glucagon secretion and the absence of UCP2 impairs α-cell function [16].

The dysregulation of uncoupling proteins (UCPs), which translocate protons into the mitochondrial matrix resulting in heat generation without ATP synthesis [17], may contribute to the pathogenesis of obesity. It has been proposed that carriers of the exon-8 insertion allele in the UCP2 gene may have a greater risk of developing obesity [18]. Papazoglou et al. have found no association between UCP2 ins/del polymorphism and morbid obesity [19]. No association between UCP2-45 bp I/D and obesity was found in a Chinese population [20]. It has been shown that the UCP gene cluster variation may not be useful predictor for type 2 diabetes mellitus (T2DM) risk assessment [21]. In the current study, we aimed to evaluate the possible association between UCP 45-bp I/D and MeS in a sample of Iranian population.

## Materials and methods

This case-control study was done on 151 patients with and 149 without MeS which we used in previous studies [4,5]. MeS was defined using the National Cholesterol Education Program Adult Treatment Panel III (NCEP ATP III) criteria [22] as described previously [23]. Ethical approvals for recruitment were obtained from local Ethics Committee of Zahedan University of Medical Sciences, and informed consent was obtained from all individuals. The data included weight, height, waist circumference, systolic and diastolic blood pressure; blood levels of glucose, triglycerides, total cholesterol, HDL cholesterol and LDL cholesterol were collected as described previously [23-25]. Blood samples were collected in EDTA-containing tubes and genomic DNA were extracted using salting out method as described previously [26].

**Figure 1:**
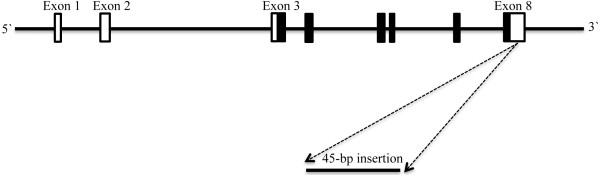
**Map of the human UCP2 gene.** Exons 1–8 are numbered and represented by black (coding exons) and white boxes (3′ and 5′ UTRs). Position of the 45-bp insertion/deletion in exon 8 in the 3′ UTR is designated.

**Figure 2:**
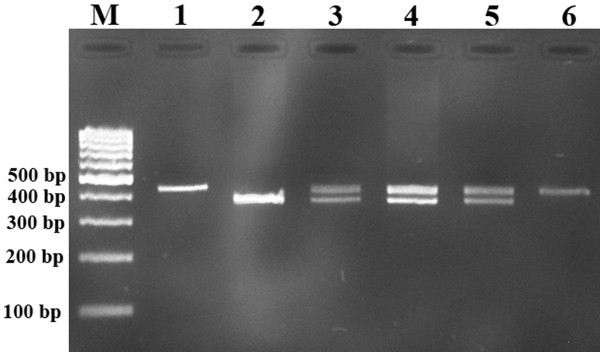
**Representative PCR products resolved by agarose gel electrophoresis to detect the presence or absence of the 45 bp I/D of UCP2 gene.** M, DNA marker. Lanes 1, 6: I/I; lane 2: D/D; lanes 3,4,5: I/D.

Position of the 45-bp I/D polymorphism of UCP2 is shown schematically in Figure 1. The forward and reverse primers for detection of 45-bp ins/del polymorphism were 5′-TCTGGCTGAACTTTCCAA-3′ and 5′-TTCATGCCCTCCTTTCTC-3′, respectively. PCR was performed by using commercially available PCR premix (AccuPower PCR PreMix; BIONEER, Daejeon, Korea) according to the manufacturer’s instructions. Briefly, 1 μL template DNA (~100 ng/mL), 1 μL of each primer (10 pmol/mL), and 17 μL DNase-free water were added to AccuPower PCR PreMix. Amplification was done with an initial denaturation step at 95°C for 5 min, followed by 30 cycles at 95°C for 30 s, 60°C for 30 s, and 72°C for 23 s with a final extension at 72°C for 10 min. The amplified PCR product was resolved on 2% agarose gel. The PCR products for insertion and deletion alleles were 428-bp and 383-bp, respectively (Figure 2). Random samples were regenotyped to verify the accuracy of genotyping. We found no genotyping mistake.

### Statistical Analysis

The differences between the categorical and contentious data were assessed by chi-square and independent sample t-tests, respectively. The association between genotypes and metabolic syndrome was assessed by computing the odds ratio (OR) and 95% confidence intervals (95% CI) from logistic regression analyses. P-value less than 0.05 were considered statistically significant. The data analysis was performed using the SPSS18 software. According to our findings, sample power was calculated by using STATA 10 software.

## Results

A total of 300 subjects including 151 MeS patients and 149 subjects without MeS were recruited in the study. The demographic and biochemical characteristics of the study participants are shown in Table 1. There were no significant differences between the groups regarding sex and age (p > 0.05). As shown in Table 2, our finding showed that the 45-bp I/D polymorphism of UCP2 decreased the risk of MeS in codominant (OR = 0.56, 95% CI = 0.34-0.92, p = 0.020 D/I vs D/D) and dominant (OR = 0.54, 95% CI = 0.34-0.86, p = 0.009; D/I + I/I vs D/D) inheritance model tested (Table 2). The I allele is associated with decreased risk of MeS (OR = 0.62, 95% CI = 0.44-0.90, p = 0.011) in comparison to D allele.

**Table 1:** Demoraphic, clinical and biochemical characteristics of individuals with and without metabolic syndrome (MeS)

	**Metabolic syndrome**		**p**
	**Yes**	**No**	
Sex (M/F)	45/104	50/101	0.901
Age (year)	43.53 ± 11.96	41.98 ± 14.65	0.382
Height (cm)	160.21 ± 9.54	161.33 ± 10.24	0.330
Weight (kg)	71.07 ± 16.48	66.35 ± 14.19	0.009
BMI (kg/m^2^)	27.59 ± 5.40	25.49 ± 4.89	0.001
Waist circumference (cm)	95.36 ± 15.54	90.19 ± 12.87	0.002
FBG (mg/dL)	96.51 ± 25.99	94.25 ± 31.27	0.459
Triglycerides (mg/dL)	184.03 ± 110.81	141.66 ± 67.23	<0.001
Total cholestrol (mg/dL)	200.03 ± 45.74	190.91 ± 39.09	0.065
HDL-C (mg/dL)	43.05 ± 9.06	45.61 ± 7.70	0.019
LDL-C (mg/dL)	120.21 ± 44.76	116.78 ± 34.61	0.460
SBP (mmHg)	122.85 ± 17.90	117.49 ± 21.57	0.020
DBP (mmHg)	77.32 ± 13.51	73.95 ± 13.61	0.032

FBG, fasting blood glucose; SBP, systolic blood pressure; DBP, diastolic blood pressure.

**Table 2:** Association of the 45-bp I/D polymorphism of UCP2 gene in individulas with and without metabolic syndrome (MeS)

**UCP2 (45-bp I/D)**	**MeS (yes)**	**MeS (No)**	^**a**^**OR (95% CI)**	**p**	**Study power %**
Codominant					
D/D	90 (59.6)	67 (45.0)	1.00	-	68
D/I	50 (33.1)	65 (43.6)	0.56 (0.34-0.92)	0.020	42
I/I	11 (7.3)	17 (11.4)	0.47 (0.21-1.08)	0.074	18
Dominanat					
D/D	90 (59.6)	67 (45.0)	1.00	-	68
D/I + I/I	61 (40.4)	82 (55.0)	0.54 (0.34-0.86)	0.009	
Recessive					
D/D + D/I	140 (92.7)	132 (88.6)	1.00	-	18
I/I	11 (7.3)	17 (11.4)	0.60 (0.27-1.34)	0.213	18
Alleles					
D	230 (76.2)	199 (66.8)	1.00	-	70
I	72 (23.8)	99 (33.2)	0.62 (0.44-0.90)	0.011	70

^a^Adjusted for sex and age.

The genotype distribution of 45-bp I/D polymorphism of UCP2 in subjects with and without MeS were in Hardy Weinberg equilibrium (HWE) (χ2 = 1.173, p = 0.278, χ2 = 0.042, p = 0.837, respectively).

## Discussion

In the present study, we analyzed the possible association 45-bp I/D polymorphism of UCP2 gene and MeS in a sample of Iranian population. The UCP2 45-bp I/D was associated with MeS, so that the frequency distribution of I/D genotype as well as I allele were significantly lower in cases than that of controls. Metabolic syndrome is a combination of risk factors for cardiovascular disease (CVD) and T2DM. These factors include hyperglycemia, hypertension, dyslipidemia (high level of triglyceride and low HDL-cholesterol), and obesity (particularly with abdominal localization) [23,27]. Prevalence of MeS varies globally and depends in part on lifestyle, sex, age and ethnicity [23,28].

Some studies have found no associations between UCP2 45-bp I/D polymorphism and obesity, resting energy expenditure, BMI and insulin secretion [29-32]. Though, in some studies, the I-allele of UCP-2 has been found to be associated with development of obesity [18,33-35].

Oguzkan-Balci et al. [36] have found That UCP I/I genotype as well as I allele was associated with childhood obesity and related metabolic disorders.

Papazoglou et al. [19] have found no association between UCP2 45-bp I/D polymorphism and morbid obesity. They found that this polymorphism has effect on weight loss in metabolically healthy subjects so that individuals with I-allele had significantly greater reduction in body mass index (BMI) and fat-free mass as well as a slight significant improvement in the homeostatic model assessment index. No association was found between UCP2 45-bp I/D and obesity in a Chinese population [20] as well as Italian Caucasians [37].

Crispim et al. [38] investigated the -866G/A (rs659366), Ala55Val (rs660339) and 45 bp I/D polymorphisms in the UCP2 gene in diabetes mellitus. They found that the haplotype [A Val I] appears to be an important risk factor associated with proliferative diabetic retinopathy in both type 1 and 2 diabetic groups.

It has been reported that UCP2 I/D heterozygous decreased the risk of end-stage renal disease (ESRD) [39]. Mitchell et al. [40] have found no association between UCP2 I/D polymorphism and neural tube defects (NTDs). While, Wang et al. [41] proposed that this variant might be a potential genetic risk factor for NTDs.

It has been proposed that impaired adipose tissue expression of UCP2 may play a role in the pathophysiology of obesity [42]. To date, very little is known about the biological effects of the UCP2 45-bp I/D polymorphism, although its location in the 3′UTR of exon 8 suggests its potential involvement in mRNA processing or in transcript stability. It has been reported that UCP2 45-bp I/D polymorphism had no apparent effect on skeletal muscle UCP2 mRNA levels in 22 randomly chosen Pima Indians [43]. Wang et al. have reported that 3′UTR I/D variant had no impact on adipose mRNA levels [44]. Esterbauer et al. showed that the ratio of inserted to deleted mRNA expression was highly variable in the adipose tissue of subjects heterozygous for 45-bp I/D. The findings suggested an independent role for the 3′UTR I/D variant in mRNA stability [45].

One of the limitations of the present study is relatively small sample size. Consequently, analysis according to MeS components was not possible.

In conclusion, our findings showed that the 45-bp I/D polymorphism of UCP2 was associated with decreased risk of MeS. Larger studies with different ethnicity are required to validate our findings.

## Competing interests

No competing financial interests.

## Authors’ contributions

MH designed the study, supervised the study, analyzed the data and drafting the manuscript. HR collected the data and carried out the laboratory studies. MAK collected the data, and reviewed the manuscript. MT analyzed the data and reviewed the manuscript. All authors read and approved the final manuscript.
